# Effect of Antimicrobial Compounds on the Survival and Pathogenic Potential of Acid-Adapted *Salmonella* Enteritidis and *Escherichia coli* O157:H7 in Orange Juice

**DOI:** 10.3390/antibiotics14040335

**Published:** 2025-03-24

**Authors:** Maria Belén Bainotti, Pilar Colás-Medà, Inmaculada Viñas, Isabel Alegre

**Affiliations:** Postharvest Biology and Technology Unit, Department of Food Technology, Engineering and Science, University of Lleida—AGROTECNIO-CERCA Centre, Av. Rovira Roure 191, 25198 Lleida, Spain; belen.bainotti@udl.cat (M.B.B.); pilar.colas@udl.cat (P.C.-M.); inmaculada.vinas@udl.cat (I.V.)

**Keywords:** coumaric acid, nisin, orange juice, acid adaptation, gastrointestinal simulation, foodborne pathogens

## Abstract

Background: The consumption of unpasteurized fruit juices poses a food safety risk due to the survival of pathogens such as *Salmonella* Enteritidis and *Escherichia coli* O157:H7. Methods: This study evaluated natural antimicrobials (nisin, coumaric acid, citral, sinapic acid, and vanillin) in orange juice as a strategy to ensure the control of these pathogens during the preservation of the non-thermally treated juices. Results: The addition of nisin, coumaric, or citral did not alter the juice’s physicochemical characteristics, ensuring product quality. Nisin (1–2 mL/L), coumaric acid (0.25–0.5 g/L), and citral (0.25–0.5 mL/L) were the most effective in reducing bacterial populations. The antimicrobial activity of the most effective compounds was then tested against both acid-adapted and non-acid-adapted bacteria in refrigerated juice, applying Weibull and linear decay models to assess bacterial inactivation. Non-acid-adapted *S.* Enteritidis showed a rapid 5 log reduction after 30 h of refrigeration with the highest nisin dose, while the acid-adapted strain exhibited a smaller reduction (2 and 1.5 log units for 1 and 2 mL/L, respectively). Citral was effective but excluded due to solubility and aroma concerns. Non-acid-adapted *E. coli* O157:H7 showed a 5 log reduction with coumaric acid at 0.5 g/L, whereas acid-adapted strains exhibited a lower reduction (around 1.5 log units). Nisin and coumaric acid also reduced bacterial survival in gastrointestinal tract simulations. However, acid-adapted bacteria were more resistant. Conclusions: These findings highlight the potential of these antimicrobials for food safety applications, though further studies should explore their mechanisms and combinations for enhanced efficacy.

## 1. Introduction

The consumption of unpasteurized fruit juices has increased in recent years due to the perception that these products better retain their nutritional and sensory properties compared to those subjected to thermal treatments [1,2]. However, *Salmonella enterica* subsp. *enterica* serovar Enteritidis and *Escherichia coli* O157:H7 have been implicated in several outbreaks linked to untreated fruit juices [3,4]. Moreover, several studies have reported that these foodborne pathogens can persist and retain their pathogenic potential in orange juice [5,6]. As a response to these outbreaks and the associated health risks, the European Union established Regulation No. 2073/2005, which mandates no detection of *Salmonella* spp. in five samples of 25 g each, as well as limits the maximum population levels of *E. coli* (10^2^–10^3^ CFU/mL) in unpasteurized juices [7].

The growing demand for natural and minimally processed products has driven the preference for fresh, untreated juices, as consumers associate these products with higher sensory quality and a more intact nutritional profile [8]. However, this habit poses significant public health risks, as the absence of pasteurization allows the survival and possible adaptation of pathogenic microorganisms in the final product. Consequently, numerous outbreaks of foodborne illnesses linked to the consumption of unpasteurized juices have been documented, highlighting the need for alternative preservation strategies [9,10].

To mitigate these risks, the food industry has explored various microbiological control strategies, with a particular focus on the use of natural antimicrobials [11,12]. Research has demonstrated that certain plant- and microbial-derived compounds exhibit effective antimicrobial activity against foodborne pathogens, enhancing product safety without compromising sensory quality or nutritional value [13].

In this context, compounds such as essential oils, organic acids, and phenolic compounds have gained attention due to their ability to inhibit microbial growth without compromising the flavour, aroma, or nutritional content of food products [14]. Citral, a major component of citrus oils, exhibits strong antimicrobial activity against a wide range of foodborne pathogens due to its ability to disrupt microbial cell membranes [15]. Nisin, a bacteriocin produced by *Lactococcus lactis*, has been extensively studied for its effectiveness against Gram-positive bacteria, making it a valuable tool in food preservation [16]. Additionally, phenolic compounds, such as coumaric and sinapic acids, are known for their antioxidant and antimicrobial properties, which can enhance food safety while also providing potential health benefits due to their bioactive nature [17,18]. Furthermore, vanillin, commonly used as a flavouring agent, also possesses antimicrobial activity, particularly against bacteria and fungi, making it a dual-purpose additive in food systems [19].

Despite the effectiveness of these compounds, a better understanding of their effect against acid-resistant pathogens, such as *S.* Enteritidis and *E. coli* O157:H7, is still needed, particularly in real food matrices like orange juice. Therefore, the present study aimed to assess the addition of natural antimicrobials to orange juice to control two pathogens, *S.* Enteritidis and *Escherichia coli* O157:H7, while considering their ability to adapt to an acidic environment.

## 2. Results and Discussion

### 2.1. Evaluation of Different Antimicrobial Compounds and Doses Against Non-Acid-Adapted S. Enteritidis and Escherichia coli O157:H7 in Orange Juice

The present study explored the effect of adding natural antimicrobial compounds, such as citral, nisin, coumaric acid, sinapic acid, and vanillin, on the survival capability of *S.* Enteritidis and *E. coli* O157:H7 in orange juice, as well as their pathogenic potential, including their ability to survive gastrointestinal tract simulation and adhere to Caco-2 cells.

**Figure A1:**
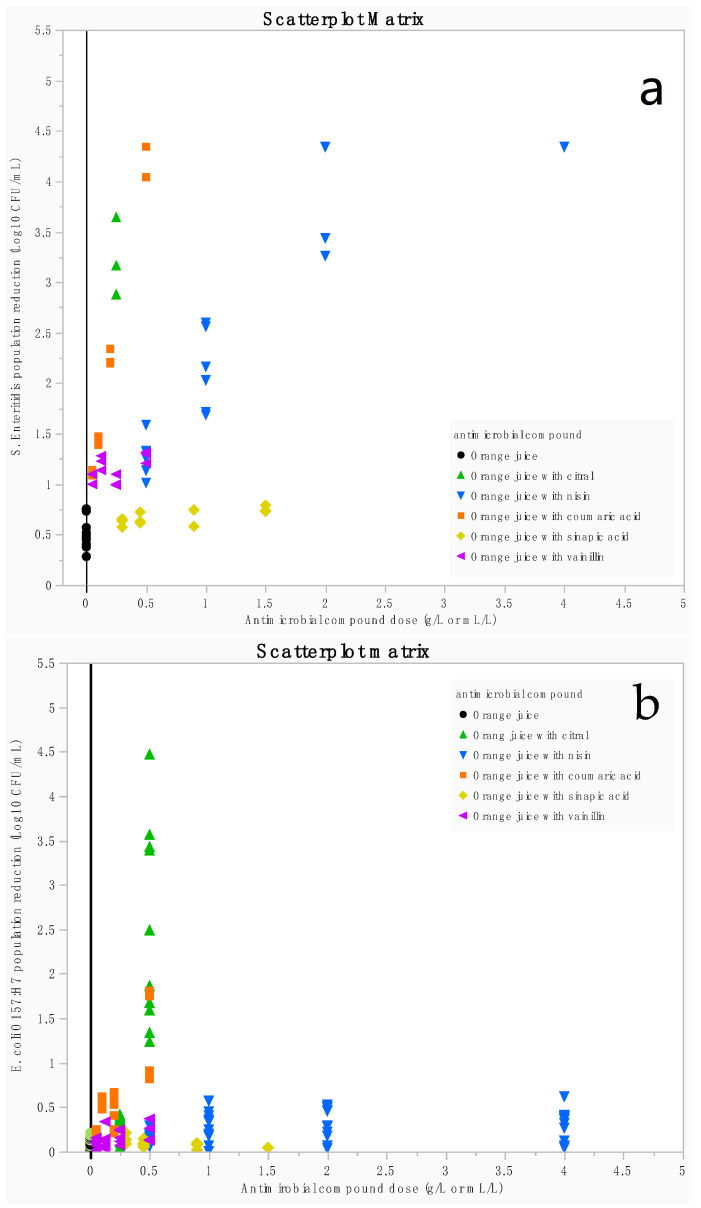
Bacterial population reductions of non-acid-adapted *S.* Enteritidis (**a**) and *E. coli* O157:H7 (**b**) after 48 h at 4 °C in orange juice with different antimicrobial compounds at different doses.

To select the most effective antimicrobial based on its ability to reduce the microbial population in the juice, the data were analysed using a scatterplot matrix (Figure A1). According to the data, as the dose of vanillin or sinapic acid increased, the population of *S*. Enteritidis or *E. coli* O157:H7 did not decrease significantly, reaching reductions of approximately 1 log unit (vanillin) and 0.5 log units (sinapic acid) after 48 h. In contrast, increasing the dose of citral, nisin, or coumaric acid resulted in greater bacterial reductions. The highest doses of nisin and coumaric acid led to the greatest reductions, achieving reductions of approximately 4 log units for both bacteria after 48 h at 4 °C. Although vanillin is commonly known as a flavouring agent, it is also widely used in food preservation and packaging [19,20,21] investigated the natural antimicrobial effect of various compounds, including vanillin, against *E. coli* O157:H7 in strawberry juice at 5 °C. To observe an immediate reduction in *E. coli* at an initial time, 5 g/L of vanillin was required. After 3 days, *E. coli* reduced from 5.0 ± 0.2 to 2.4 ± 0.1 log units, with a concentration of 2.5 g/L. Differences between our findings and those of other studies could stem from their use of much higher concentrations than those tested in our work. Regarding sinapic acid, extensive research has been conducted on its origin, extraction, characterization, and medicinal properties as a phenolic acid. While the antibacterial activity of sinapic acid has been confirmed in various studies on plant and human pathogens [18], our results did not show a strong antimicrobial effect. Engels et al. [22] confirmed sinapic acid’s antibacterial activity against multiple pathogens, including *E. coli* (MIC = 0.7 g/L). Liu et al. [23] attributed the limited efficacy of sinapic acid to its low solubility and its chemical structure. They suggested that the outer lipid layer of the Gram-negative bacterial cell wall could explain its relatively weak activity. Specifically, they proposed that the steric hindrance caused by the relatively large size of sinapic acid, combined with its higher hydrophilicity due to OH and methoxy groups, may reduce effective interactions with *E. coli*. These structural factors could also contribute to the observations in our study.

Once citral, nisin, and coumaric acid were selected as the compounds that presented the major antimicrobial effect, the next step was to determine the appropriate doses of these antimicrobials against non-acid-adapted *S.* Enteritidis and *E. coli* O157:H7 in orange juice under refrigeration. The populations of *S.* Enteritidis and *E. coli* O157:H7 after juice inoculation were 5.0 ± 0.1 log CFU/mL and 5.1 ± 0.1 log CFU/mL, respectively. Bacterial population reductions after 48 h in orange juice and orange juice with antimicrobials are shown in Table 1. For citral, it was decided to continue experimentation with both doses (0.25 and 0.5 mL/L), as there were statistically significant differences between them. The decision to test two doses of citral was based on preliminary studies, which revealed that higher doses resulted in incomplete solubilization in orange juice. To address these methodological challenges, some researchers are exploring encapsulation strategies for essential oils, such as citral, to incorporate them into fruit juices and thereby enhance their stability and solubility [24,25]. For coumaric acid, the higher doses (0.25 and 0.5 g/L) were selected, as they had the greatest effect on bacterial population reduction. The lower doses (0.05 and 0.1 g/L) barely reduced the population of *E. coli* O157:H7 (less than 0.5 log units of reduction). Lastly, although nisin did not show significant reductions in *E. coli* O157:H7 (reductions of less than 0.4 ± 0.2 log units), its addition to the juice resulted in a population reduction for *S.* Enteritidis. The 1 and 2 mL/L doses of nisin were selected because they showed the greatest *S.* Enteritidis population reduction, and there were no significant differences between the 2 mL/L dose and the highest dose (4 mL/L).

### 2.2. Evaluation of Antimicrobial Activity of the Most Effective Compounds at Different Doses Against Acid-Adapted and Non-Acid-Adapted Bacteria in Orange Juice

One of the first assays carried out aimed to evaluate whether the addition of these compounds to juice modified the physicochemical characteristics of the food matrix. The physicochemical characteristics of the orange juice and the orange juice with the addition of different antimicrobials were evaluated (Table 2). The orange juice had a pH of 3.51 ± 0.02, total soluble solids (TSS) of 11.6 ± 0.1 °Brix, a titratable acidity (TTA) of 7.0 ± 0.26 g citric acid/L, an a_w_ of 1.000 ± 0.002, and total phenolic content (TPC) of 528.39 ± 25.22 mg gallic acid/mL. Antioxidant capacity was determined by the DPPH and the FRAP methods, and the results were 1200 ± 3.85 mg ascorbic acid/L and 977.98 ± 27.55 mg ascorbic acid/L, respectively. There were no significant differences in the values of all the physicochemical characteristics evaluated between the control juice and the juice samples with added antimicrobials. Several researchers evaluated whether the addition of natural antimicrobial compounds modified the physicochemical characteristics of fruit juice both at the time of addition and throughout storage at low temperatures (≈5 °C) [14,26,27]. As in this study, pH, TSS, or TTA were not modified. However, and unlike the results obtained, the addition of antimicrobial compounds produced a significant increase in TPC and antioxidant capacity. Cassani et al. [28] observed that adding vanillin to strawberry juice also increased the TPC and antioxidant capability when compared to untreated strawberry juice. Although the increase in TPC and antioxidant capacity is expected because some of these compounds are naturally phenols or antioxidant compounds, the differences could be due to the added concentrations. The researchers used a higher vanillin concentration (1.8 g/L) than the one evaluated in this work (maximum of 0.5 g/L), which would lead to the observable increase in TPC in the samples. Phenolic and antioxidant compounds, in general, are considered good additives to increase the nutritional value of food products [29].

**Figure 1:**
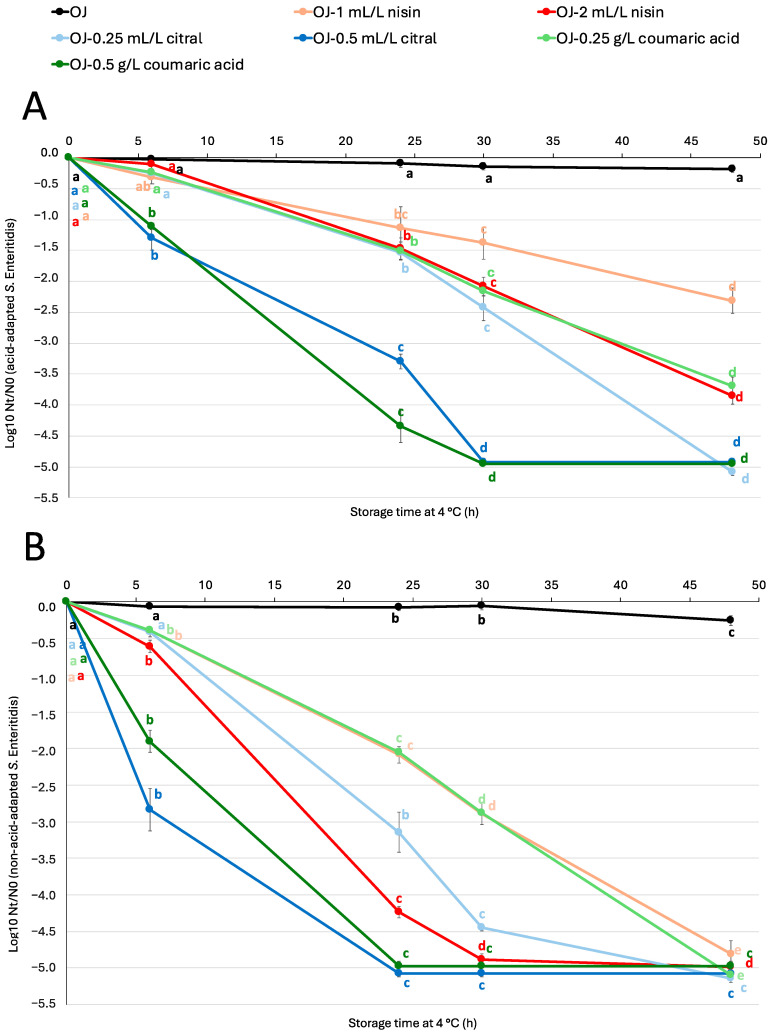
Logarithmic reduction of acid-adapted (**A**) and non-acid-adapted (**B**) *S*. Enteritidis (Log_10_ N_t_/N_0_) in orange juice and in orange juice with different doses of antimicrobial compounds during storage at 4 °C. Points represent the mean of six repetitions, and error bars represent standard error of the mean. Different letters represent significant differences over storage time, according to analysis of variance (ANOVA) and Tukey’s test (*p* < 0.05). OJ: orange juice.

**Figure 2:**
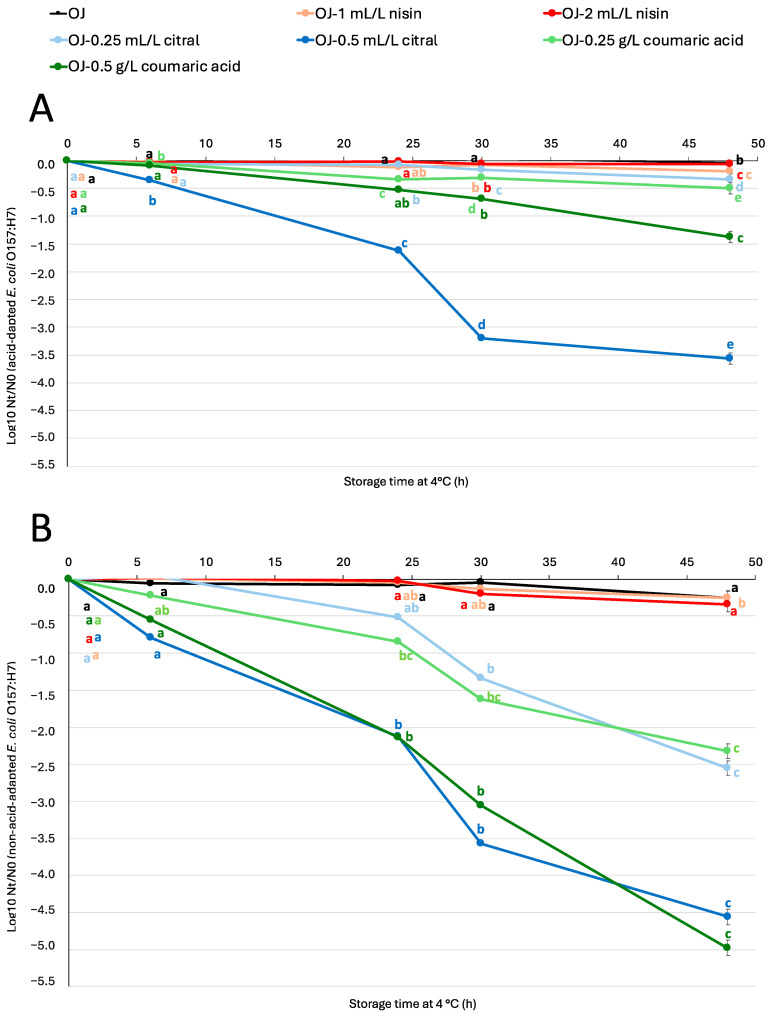
Logarithmic reduction of acid-adapted (**A**) and non-adapted (**B**) *E. coli* O157:H7 (Log_10_ N_t_/N_0_) in orange juice and in orange juice with different doses of antimicrobial compounds during storage at 4 °C. Points represent the mean of six repetitions, and error bars represent standard error of the mean. Different letters represent significant differences over storage time, according to analysis of variance (ANOVA) and Tukey’s test (*p* < 0.05). OJ: orange juice.

Figure 1 and Figure 2 show the logarithmic reduction of both acid-adapted and non-acid-adapted *S.* Enteritidis (Figure 1) and *E. coli* O157:H7 (Figure 2) populations in orange juice with and without antimicrobials at two different doses. The populations of acid-adapted *S.* Enteritidis and both acid-adapted and non-acid-adapted *E. coli* O157:H7 in orange juice without antimicrobials remained constant throughout the incubation, but non-acid-adapted *S.* Enteritidis showed a slight reduction (less than 0.5 log units) after 48 h. In contrast, in orange juice with antimicrobials, the reduction was greater compared to juice without antimicrobials in most cases. In juices with antimicrobials, a trend was observed: the higher the dose, the greater the bacterial reduction.

The antimicrobial peptide nisin has been widely used to prevent the contamination of food products by Gram-positive pathogens, including *Listeria monocytogenes* [27]. However, studies have shown that certain serovars of *L. monocytogenes* exhibit tolerance or resistance to nisin at doses that would typically be lethal [30,31]. In our study, we evaluated the effectiveness of this antimicrobial against two different pathogens to determine whether nisin could be a viable alternative for controlling foodborne bacteria beyond *L. monocytogenes*. Our findings revealed that non-acid-adapted *S.* Enteritidis showed a rapid reduction in the juice with the higher dose of nisin (2 mL/L), reaching nearly 5 log units after 30 h of incubation. Similar results were observed by Fernandez da Silva et al. [32], who studied the influence of environmental factors on the sensitivity of *Salmonella* Typhimurium to nisin in refrigerated orange juice. The researchers obtained results similar to those in this study, observing the highest efficiency at a lower pH (4.0) and with a bacteriocin concentration of 174 µM (approximately 0.58 g/L). When tested in orange juice, nisin caused a reduction of up to 4.1 log units in the *Salmonella* population. These results suggest that nisin could be an effective alternative for controlling *Salmonella* in food matrices, as environmental factors such as low pH and low temperature appear to enhance the sensitization of *Salmonella* cells to nisin’s bactericidal action. In contrast, the acid-adapted *S.* Enteritidis population did not decrease as much (around 2 and 1.5 log units at the same time for 1 mL/L and 2 mL/L of nisin, respectively) at the same time (30 h). However, the observed difference between acid-adapted and non-acid-adapted strains indicates that prior acid adaptation may confer cross-resistance to nisin. Regarding *E. coli* O157:H7, the addition of nisin at both different doses did not reduce the bacteria populations, with the behaviour of this microorganism being very similar to its behaviour in untreated orange juice. Guo et al. [33] investigated the impact of free nisin nano emulsions and nisin combined with fatty acids. Similar to our study, the researchers did not observe significant reductions in *E. coli* following nisin treatment. However, the combination of nisin with fatty acids in nano emulsions yielded promising results in reducing pathogen populations. This approach—combining nisin with other compounds—is both promising and innovative in enhancing its antimicrobial effectiveness against Gram-negative bacteria such as *Salmonella* and *E. coli* [34,35]. Nonetheless, our findings indicate that in some cases, such as *Salmonella*, nisin alone proved to be an effective antimicrobial additive in orange juice. In contrast, for *E. coli*, where nisin exhibited limited antimicrobial activity, combining it with another compound could be a more effective strategy.

Citral showed a significant effect on both bacteria, especially at the higher dose (0.5 mL/L citral). At this dose, there was a rapid and drastic reduction in the populations of acid-adapted *S.* Enteritidis, reaching approximately 5 log units of reduction in less than 48 h. The addition of citral to orange juice did not result in rapid reductions in *E. coli* O157:H7; instead, the populations progressively decreased. It is important to note that the addition of low doses of citral (0.25 g/L) was not effective in reducing the population of this pathogen when it was acid-adapted, with reductions of less than 0.5 log units after 48 h. These findings align with the results of Raybaudi-Massilia et al. [36], who observed that the antimicrobial activity of essential oils, such as citral, depends on the type of microorganism and environmental conditions, including pH and temperature. Specifically, Burt [37] suggested that a low food pH increases the hydrophobicity of the essential oil, enabling it to dissolve more easily into the lipid membrane of target bacteria. Friedman et al. [38] observed a greater bactericidal activity of essential oils at 37 °C compared to 4 °C, indicating that higher temperatures may enhance the effectiveness. Although our experiments were conducted at low storage temperatures (4 °C), an increase in temperature could have improved citral’s activity. Nevertheless, the strategy to combining treatment with an increase in temperature could compromise juice quality and safety. Another limitation of citral is its low solubility in aqueous matrices. Numerous studies have proposed encapsulating citral to improve its stability and solubility in liquid foods. Donsì et al. [24] reported that encapsulating essential oils in nano emulsions allows for a gradual, controlled release, maximizing pathogen contact. Orizano-Ponce et al. [39] applied an emulsification strategy using modified corn starch to improve the miscibility of citral in aqueous food matrices, achieving homogeneous dispersion and enhanced bacterial inactivation in fruit juices. Moreover, some studies have observed that combinations of essential oils have a synergistic effect that improves antimicrobial efficacy [40]. In this context, combining citral with other natural antimicrobials or processing techniques, such as high-pressure processing, could be an effective solution to overcome the resistance of adapted strains [15].

Lastly, the addition of coumaric acid to orange juice showed different effects, depending on the microorganism. For *S.* Enteritidis, high doses of coumaric acid (0.5 g/L) resulted in greater reductions, reaching 5 log units after 24 h in non-acid-adapted and after 30 h in acid-adapted populations. Similar to the behaviour observed with nisin and contrary to the behaviour with *S.* Enteritidis, the addition of coumaric acid to the juice did not drastically reduce *E. coli* O157:H7 populations. The response of the non-acid-adapted strain to coumaric acid was similar to that observed with citral at both doses. The highest reduction was observed with the higher dose, reaching almost 5 log units after 48 h. For acid-adapted *E. coli* O157:H7, the higher dose was also the most effective, although the reduction values were close to only 1.5 log units after 48 h. These findings confirm that coumaric acid effectively inhibits the growth of these pathogens and could serve as a promising natural antimicrobial alternative. The mechanism of action of coumaric acid is based on disrupting plasma membrane integrity, allowing the compound to enter the cell, where it interacts with proteins and genomic DNA, ultimately leading to cellular damage and lysis. Furthermore, as the concentration of this phenolic acid increases, its antimicrobial effectiveness also improves, a trend that has been observed in recent studies [41]. When comparing these results with those obtained by Lou et al. [42], it is evident that the concentration required to achieve a significant population reduction in our study is higher than the minimum inhibitory concentrations (MICs) established by these researchers (0.02 g/L for *S.* Typhimurium and 0.08 g/L for *E. coli*). Additionally, Lou et al. [42] reported that Gram-positive and Gram-negative bacteria exhibit different MIC values for coumaric acid, which suggests that its effectiveness is influenced by bacterial cell structure. Although both pathogens evaluated in this study are Gram-negative, they are different species with variations in their outer membrane composition, which could explain the observed differences in behaviour. Moreover, in bacteria adapted to stress conditions, such as acidic environments, defence mechanisms are activated, leading to structural modifications in the membrane and cell wall [43]. This adaptation could also explain the great survival of acid-adapted bacteria to coumaric acid in fruit juice. The ability of these microorganisms to alter their cellular structures in response to stress may contribute to their enhanced survival, even in the presence of antimicrobial compounds.

The acid adaptation observed in *S.* Enteritidis and *E. coli* O157:H7 supports the hypothesis of cross-resistance, particularly under sublethal antibacterial stress conditions, a relevant phenomenon in food safety. Studies such as Aiyedun et al. [44] have noted that exposure to acidic environments can induce the expression of stress-resistance genes, making bacteria less susceptible to antimicrobial treatments. In our study, this acid adaptation delayed population reduction in the presence of citral, requiring an increased concentration of the antimicrobial to overcome adaptive resistance. This finding is consistent with Pagnossa et al. [45], who reported that eliminating *Salmonella* strains adapted to sublethal doses of antimicrobials like cinnamaldehyde and citral required higher concentrations of alternative compounds such as linalool, highlighting bacterial adaptive capacity to essential oils. In this context, Chung et al. [40] demonstrated that a promising strategy to counter this cross-resistance in acid-adapted bacteria, such as *E. coli* O157:H7 and *Salmonella*, is the use of synergistic combinations of essential oils. This antimicrobial combination shows a synergistic effect that enhances inactivation, offering an effective solution to ensure the elimination of adapted pathogens.

The data obtained in the evaluation of antimicrobial activity of citral (0.25 and 0.5 mL/L), nisin (1 and 2 mL/L), and coumaric acid (0.25 and 0.5 g/L) were fitted using two predictive bacterial decay models (Table 3). From the modelling, different kinetic parameters were derived based on the linear model (Table 4) and the Weibull model (Table 5).

Not all data could be fitted to the predictive models. The lack of fit is due to the absence of microbial population decline, indicating a limited effect of the antimicrobial on the population or the orange juice alone. According to the linear model, the Kmax values (first-order inactivation rate constant) show that the antimicrobials, especially citral and coumaric acid at higher doses (OJ-0.5 mL/L citral and OJ-0.5 g/L coumaric acid), are more effective at reducing the populations of both pathogens. According to the Weibull model, the parameter δ (time required for the first decimal reduction) was lower in treatments with high doses of citral and coumaric acid, indicating that these compounds have a greater capability for rapid bacterial inactivation compared to nisin. Most of the values of p (shape parameter) were greater than 1, indicating that the bacterial population curves over time exhibit a concave shape.

### 2.3. Effect of Antimicrobial Compounds in the Pathogenic Potential of Acid-Adapted and Non-Acid-Adapted S. Enteritidis and E. coli O157:H7

**Figure 3:**
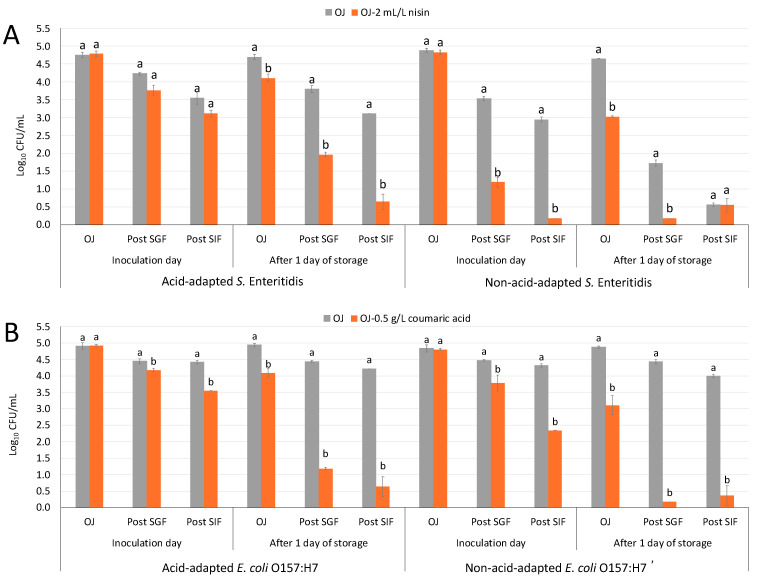
Survival population of acid-adapted and non-acid-adapted *S.* Enteritidis (**A**) and *E. coli* O157:H7 (**B**) in orange juice and orange juice with nisin (2 mL/L) or coumaric acid (0.5 g/L) following exposure to a simulated gastrointestinal tract (GIT) before and after 1 day of juice storage at 4 °C. Bars represent the mean of six repetitions, and error bars represent standard error of the mean. Different letters indicate significant differences between orange juice (grey bars) and orange juice with an antimicrobial compound (orange bars) at each sampling moment: orange juice (OJ) after exposure to simulated gastric fluid (post SGF) and after exposure to simulated intestinal fluid (post SIF) according to analysis of variance (ANOVA) and Tukey’s test (*p* < 0.05).

Although citral showed a certain effect in pathogen survival (previous results in Section 2.2), its impact on pathogenic potential was not evaluated due to both bibliographic evidence and empirical observations in the laboratory, which demonstrated that citral was not fully soluble in orange juice. Figure 3 represents the survival capability of acid-adapted and non-acid-adapted *S.* Enteritidis (A) and *E. coli* O157:H7 (B) in orange juice and orange juice with antimicrobial compounds at 4 °C following exposure to gastrointestinal tract simulation. The results demonstrated that the addition of antimicrobials, specifically nisin (2 mL/L) for *S.* Enteritidis and coumaric acid (0.5 g/L) for *E. coli* O157:H7, significantly reduced the populations of both acid-adapted and non-acid-adapted foodborne pathogens compared to untreated orange juice. Notably, non-acid-adapted strains exhibited greater susceptibility to both antimicrobials, with larger reductions in populations compared to acid-adapted strains. For non-acid-adapted *S.* Enteritidis, the initial population of 4.8 ± 0.2 log CFU/mL units in orange juice with nisin decreased dramatically to 1.2 ± 0.3 log CFU/mL after SGF exposure and reached 0.2 ± 0.0 log CFU/mL after SIF, maintaining this low level on day 1 (Figure 3A). This represents a reduction of more than 4 log units, demonstrating the strong efficacy of nisin in reducing non-adapted *S.* Enteritidis populations. In contrast, in orange juice without nisin, the same strain showed moderate reductions, from 4.8 ± 0.2 log CFU/mL to 2.9 ± 0.1 log CFU/mL after SIF, reinforcing the limited impact of orange juice alone. A similar trend was observed for non-acid-adapted *E. coli* O157:H7 when treated with coumaric acid. The initial population of 4.8 ± 0.1 log CFU/mL dropped to 3.8 ± 0.6 log CFU/mL after exposure to SGF, further reduced to 2.3 ± 0.3 log CFU/mL post SIF, and continued to decline to levels below 0.5 log CFU/mL by day 1 (Figure 3B). This marked reduction of more than 4 log units highlights the potent antibacterial effect of coumaric acid on non-acid-adapted *E. coli* O157:H7. In contrast, in orange juice without an antimicrobial, the same bacterial population only showed minor decreases, from 4.9 ± 0.1 log CFU/mL to 4.0 ± 0.1 log CFU/mL after SIF exposure, indicating limited bacterial inactivation without the antimicrobial.

Overall, the survival of both bacteria in orange juice without antimicrobials was lower after exposure to SIF compared to SGF, but the reductions remained inferior to those observed in juice containing antimicrobials. These findings confirm the enhanced antimicrobial effectiveness of nisin and coumaric acid, particularly against non-acid-adapted strains, and highlight their potential as natural food safety interventions. The use of natural compounds with inhibitory effects on pathogenicity has emerged as a promising approach to controlling foodborne pathogens. This strategy aims to interfere with virulence factors that facilitate bacterial adhesion, colonization, invasion of the host, and immune system evasion [46]. Studies have demonstrated that the use of beneficial bacteria (*Lactobacillus*, *Bifidobacterium*), chemical molecules (such as chalcone), and biological compounds (such as mucin) can inhibit the virulence of both Gram-positive and Gram-negative pathogens, providing new alternatives for infection prevention [47].

Acid-adapted strains were more resilient to antimicrobial treatments, though notable reductions were still observed. Acid-adapted *S.* Enteritidis artificially inoculated in orange juice with nisin showed a population reduction from 4.8 ± 0.2 log CFU/mL to 3.1 ± 0.2 log CFU/mL after SIF and went down to below 0.6 log CFU/mL by day 1 (Figure 3A). Similarly, the acid-adapted *E. coli* O157:H7 population in orange juice with coumaric acid reduced from 4.9 ± 0.1 log CFU/mL initially to 3.6 ± 0.0 log CFU/mL after SIF exposure, with a further decline to below 0.6 log CFU/mL by day 1 (Figure 3B). These results demonstrate the antimicrobial effect and reduction in pathogenic potential observed when compounds such as nisin or coumaric acid are present in acidic conditions, both in SGF and orange juice. However, the exact mechanism by which this interaction enhances antimicrobial activity, as well as how acid-adapted bacteria can sometimes evade this effect, remains unclear.

One of the main challenges in using nisin against Gram-negative bacteria is its difficulty in crossing the outer membrane due to its molecular size and the structure of the bacterial cell wall. *S.* Enteritidis, as a Gram-negative bacillus, possesses an outer membrane rich in lipopolysaccharides (LPSs), which form a highly selective barrier that restricts the passage of antibiotics, hydrophobic compounds, detergents, and dyes [48]. This barrier is stabilized by divalent cations such as Ca^2^⁺ and Mg^2^⁺, which contribute to the structural rigidity of the membrane. However, in an acidic environment, the increased concentration of protons (H⁺) competes with these cations for binding sites on the LPS, leading to their displacement and weakening the integrity of the outer membrane [49]. As a result, the LPS structure becomes more permeable, exposing the inner phospholipid layer and facilitating the entry of certain antimicrobials, including nisin [50]. This suggests that under low-pH conditions, nisin may be more effective against *S.* Enteritidis, as the weakened outer membrane would allow greater access and bactericidal activity. Nevertheless, acidic environments, such as orange juice (pH 3.51 ± 0.02), can induce the acid tolerance response (ATR) in *Salmonella* spp., a mechanism that protects the bacteria from more extreme stress conditions and may enhance their virulence [51]. We think that the presence of nisin may interfere with ATR development, making both acid-adapted and non-acid-adapted *S.* Enteritidis equally vulnerable to acid stress. When evaluating the results of our study in light of this information, the increased inhibition of *S.* Enteritidis in the presence of nisin could be explained by the weakening of the outer membrane, making *Salmonella* cells more susceptible to the antimicrobial. However, this effect may not be observed in *E. coli* in the experiments where all antimicrobial compounds were evaluated due to potential differences in outer membrane composition and permeability. While both *S.* Enteritidis and *E. coli* possess LPSs, variations in LPS structure, membrane protein composition, and regulatory responses to acidic stress could influence their susceptibility to nisin. Additionally, *E. coli* might have a more robust ATR or alternative mechanisms that counteract membrane destabilization, preventing nisin from exerting the same antimicrobial effect observed in *S.* Enteritidis. Further investigation is needed to determine the specific factors that contribute to these differences.

Similarly, the reduction in the pathogenic potential of *E. coli* O157:H7 following the addition of coumaric acid indicates that its antimicrobial effect is not affected by prior acid adaptation. Yuan et al. [52] observed that while cross-resistance existed between tolerance to certain antimicrobials (such as carvacrol and cinnamaldehyde) and resistance to heat or desiccation, it was not associated with acid resistance. These findings align with our observations, where a decrease in the pathogenic potential of *E. coli* O157:H7 suggests that coumaric acid exerts its antimicrobial effect independently of previous acid adaptation, reinforcing its potential as a control strategy for this pathogen.

Lastly, in the adhesion capability assay, the results showed no significant differences in adhesion capability between acid-adapted and non-acid-adapted bacteria in the same food matrix at the inoculation time (Table 6). The adhesion capacity data after 1 day of storage at 4 °C were not determined, as the low pathogen populations in some treatments resulted in an insufficient bacterial concentration to expose Caco-2 cells to a comparable bacterial load. Yin et al. [53] evaluated the adhesion capacity of *S.* Typhimurium to the same cell line used in this study after 24 h at 4 °C in grapefruit juice, supplemented with two common citrus compounds, naringenin and naringin. The researchers observed adhesion percentages lower than 12% and an 80% reduction in adhesion in antimicrobial-supplemented juice compared to the control. Their study demonstrated that this significant decrease in adhesion capacity was strongly influenced by the low pH and the addition of the antimicrobial, a finding that may be reflected in our results. However, to confirm this with certainty, lower antimicrobial concentrations or higher initial bacterial inoculum levels should be tested to establish more precise correlations. Therefore, both antimicrobials could act synergistically with the low pH of orange juice to inhibit the survival and pathogenic potential of these pathogens. A deeper understanding of the mechanisms of action of nisin and coumaric acid against Gram-negative bacteria could help expand their application as antimicrobial agents and enhance their use in the food industry.

## 3. Materials and Methods

### 3.1. Orange Juice with Antimicrobial Compounds

Commercial pasteurized orange juice obtained from a local supermarket in Lleida (Catalonia, Spain) was used as a food matrix to determine the effectiveness of certain organic compounds with antimicrobial activity. The organic compounds evaluated were as follows: citral (Sigma-Aldrich; San Luis, MO, USA), nisinA (White NisinA^®^, Handary; Fleurus, Belgium), p-coumaric acid (Sigma-Aldrich; San Luis, MO, USA), sinapic acid (Sigma-Aldrich; San Luis, MO, USA), and vanillin (Sigma-Aldrich; San Luis, MO, USA). Antimicrobial compounds were added at different concentrations to commercial orange juice. Orange juice without added compounds was included as a control. Samples were stored in 20 mL aliquots in sterile bottles at −20 °C until use.

### 3.2. Bacterial Culture and Media

In this work, two foodborne pathogens, *S.* Enteritidis (CECT 4300) and *E. coli* O157:H7 (NCTC 12900), were analysed. To prepare a fresh culture of each bacterium, a single colony grown in Tryptone Soy Agar (TSA; Biokar; Allonne, France) for 24 ± 1 h at 37 ± 1 °C was inoculated in 100 mL of Tryptone Soy Broth (TSB; Biokar; Allonne, France) for 20 ± 1 h at 37 ± 1 °C. The incubation time was selected to obtain cells at the end of the logarithmic phase of growth. After foodborne pathogen incubation, 20 mL of suspension was centrifugated at 9800× *g* for 10 min at 25 °C and re-suspended in Saline Solution (SS; 8.5 g/L NaCl; VWR; Radnor, PA, USA). Concentrations were verified by plating appropriate tenfold dilutions in Saline Peptone (SP; 8.5 g/L NaCl; VWR; Radnor, PA, USA, and 1 g/L peptone; Biokar; Allonne, France) onto TSA. Plates were incubated for 24 ± 2 h at 37 ± 1 °C.

To obtain acid-adapted bacteria, fresh bacterial cultures (50 µL) were inoculated in 20 mL of flasks with TSB with 1% glucose (TSB without glucose; Sigma-Aldrich; San Luis, MO, USA, and 10 g/L glucose, D-Glucose anhydrous; Fisher Scientific; Loughborough, UK) media. To obtain non-adapted bacteria, fresh bacteria culture was inoculated in 20 mL of TSB without glucose. All flasks were inoculated at a concentration of 10^3^ CFU/mL and incubated at 37 ± 1 °C for 24 ± 2 h. The initial and final pH values of each culture condition were measured using a pH meter and taken as a control parameter. For acid-adapted bacteria, the initial and final pH values were 7.0 ± 0.1 and 4.7 ± 0.1, respectively. For non-acid-adapted bacteria, the initial and final pH values of the medium were 7.0 ± 0.1 and 6.6 ± 0.1, respectively. Populations were counted by plating appropriate tenfold dilutions in SP onto TSA. Plates were incubated at 37 ± 1 °C for 24 ± 2 h.

### 3.3. Antimicrobial Activity of Organic Compounds Against S. Enteritidis and E. coli O157:H7

Firstly, the effects of different organic compounds against non-acid-adapted bacteria in orange juice were evaluated (Section 3.3.1). Secondly, the antimicrobials that showed the best effectiveness were evaluated against acid-adapted and non-adapted bacteria in orange juice during refrigerated storage (Section 3.3.2). Finally, a single antimicrobial was selected for each microorganism to determine its effect on the pathogenicity of the two bacteria, acid-adapted and non-acid-adapted (Section 3.3.3).

#### 3.3.1. Selection of the Most Effective Antimicrobial Compounds and Doses Against Non-Acid-Adapted Bacteria in Refrigerated Orange Juice

Based on a literature review and with the aim of determining the minimum concentration of each compound necessary to inhibit the growth of each pathogen, the following concentrations were added to orange juice: 0.25 and 0.5 mL/L of citral; 0.5, 1, 2, and 4 mL/L of nisin; 0.05, 0.1, 0.25, and 0.5 g/L of coumaric acid; 0.3, 0.45, 0.9, and 1.5 g/L of sinapic acid; and 0.05, 0.125, 0.25, and 0.5 g/L of vanillin. In each trial, a sample of orange juice without added compounds was included as a control (OJ). Before inoculation, all enriched orange juices were tempered a 4 °C.

Each sample was inoculated with 200 μL of foodborne pathogen, obtained after centrifuging a 24 h culture grown in TSB, to achieve an initial concentration of 5 log units in fruit juice. Samples were stored at 4 °C during 48 h, and population monitoring was conducted after inoculation and at the end of storage. At each sampling time, a bacterial counting method was performed on TSA plates and incubated at 37 ± 1 °C for 24–48 h.

#### 3.3.2. Evaluation of Antimicrobial Activity of the Most Effective Compounds at Different Doses Against Acid-Adapted and Non-Acid-Adapted Bacteria in Orange Juice

First of all, the antimicrobials that showed greater effectiveness according to the results of Section 3.3.1 were selected: citral (0.25 and 0.5 mL/L), nisin (1 and 2 mL/L), and coumaric acid (0.25 and 0.5 g/L). For the determination of the physicochemical characteristics of each sample, pH was measured using a pH meter (GLP22 pH-meter model; Crison Instruments S.A; Alella, Spain) equipped with an electrode (2 PORE F TEMP BNC electrode; XS Instruments; Carpi, Italy). TSS were measured at room temperature using a digital refractometer (Atago Co. Ltd; Bellevue, WA, USA), and results were expressed in °Brix, with the “±” symbol indicating the standard deviation (SD). To assess TTA, 10 mL of juice was titrated with 0.1 N NaOH until reaching a pH of 8.1, and results were expressed as mg of the predominant organic acid/L ± SD. TPC and antioxidant activity were determined following the method described by Nicolau-Lapeña et al. [54]. TPC results were expressed as mg of gallic acid equivalents (GAE)/mL. Antioxidant activity was evaluated using the DPPH and FRAP methods. Results were expressed as mg of ascorbic acid/L ± SD.

Twenty mL of acid-adapted and non-acid-adapted *S.* Enteritidis and *E. coli* O157:H7 cultures, grown in TSB with 1% glucose and TSB without glucose, respectively, was centrifuged at the same conditions previously mentioned. Then, the pellets were resuspended in the same volume of sterile water to obtain a suspension free of media. Sterile flasks with 20 mL of orange juice and orange juice with antimicrobial previously tempered a 4 °C were inoculated with each strain to obtain an estimated initial concentration of 10^5^ CFU/mL and then stored at 4 °C. The monitoring of the bacterial population in the samples was carried out at 0, 6, 24, 30, and 48 h by plating appropriate tenfold dilutions in SP onto TSA media. Plates were incubated at 37 ± 1 °C for 24 ± 2 h.

#### 3.3.3. Effect of Antimicrobial Compounds in the Pathogenic Potential of Acid-Adapted and Non-Acid-Adapted *S*. Enteritidis and *E. coli* O157:H7

Based on the results of Section 3.3.1 and Section 3.3.2, the most promising antimicrobial compound and dose were nisin at 2 mL/L and coumaric acid at 0.5 g/L against *S.* Enteritidis and *E. coli* O157:H7, respectively. The pathogenic potential of acid-adapted and non-acid-adapted bacteria was evaluated in terms on their ability to survive a gastrointestinal tract simulation and the proportion of cells capable of subsequently adhering to a differentiated Caco-2 cell monolayer. Bacterial counts were performed at the following different sampling times: immediately after inoculation (OJ), after exposure to simulated gastric fluid (post SGF), and after exposure to simulated intestinal fluid (post SIF). The same procedure was repeated after 1 day of storage at 4 °C. Additionally, adhesion capability was assessed after gastrointestinal tract simulation on the day of inoculation and again after 1 day of storage at 4 °C.

Gastrointestinal tract simulation assay was conducted following the Minekus et al. [55] method, with some modifications. At each sampling point, 5 mL of inoculated orange juice or antimicrobial enriched orange juice was placed into a sterile flask. Subsequently, 9.8 mL of Simulated Gastric Fluid (SGF, pH 3.0) was added, and the mixture was homogenized, adjusting the pH to 3.0 with hydrochloric acid (HCl; 1 mol/L; Panreac S.A; Barcelona, Spain). Samples were incubated at 37 ± 1 °C for 2 h. After the incubation, the pH was measured again, and a bacterial count was performed using 0.5 mL per duplicate of each sample used (post SGF). Then, 19.7 mL of simulated intestinal fluid (SIF, pH 7.0) was added and homogenized, adjusting the pH to 7.0 with sodium hydroxide (NaOH; 1 mol/L; VWR; Radnor, PA, USA). Samples were incubated again at 37 ± 1 °C for 2 h. The pH of the final mixture was measured, and duplicate 0.5 mL samples were collected for bacterial count (post SIF). The counting method for each sampling point was performed as described previously.

A human colon adenocarcinoma cell line (Caco-2; ECACC 86012202) was used to assess adhesion capability following the methodology described by Ortiz-Solà et al. [56], with modifications. To ensure a similar multiplicity of infection (MOI), an aliquot of each sample after gastrointestinal tract simulation was centrifuged at 9800× *g* for 10 min at 25 °C and then resuspended in 6 mL of Dulbecco’s Modified Eagle Medium (DMEM; Hyclone VWR; Radnor, PA, USA) supplemented with 15% heat-inactivated foetal bovine serum (FBS; Hyclone VWR; Radnor, PA, USA). Suspensions were quantified by plating them on appropriate selective media to determine initial bacterial concentrations (Xylose Lysine Deoxycholate agar, XLD; Biokar; Allonne, France, for *S.* Enteritidis and MacConkey Sorbitol agar, CT-SMAC; Biokar; Allonne, France, for *E. coli* O157:H7). Two wells of cells were used for the adhesion assay, with each well inoculated with 1 mL of the bacterial sample. The plates were incubated for 1 h at 37 °C, 5% CO_2_, and 95% humidity. After incubation, the media were aspirated, and the monolayers were washed twice with Sterile Phosphate-Buffered Saline (PBS, Gibco, Thermo Scientific; Waltham, MA, USA) to remove non-adhered bacteria. The cells were then lysed to release the adhered bacteria using 1 mL of 0.1% (*v*/*v*) Triton-X100 (Sigma-Aldrich; San Luis, MO, USA) in PBS, and the collected lysate was plated onto nutritive media, TSA. Plates were incubated for 24 h at 37 °C to determine the number of adhered bacteria.

### 3.4. Data Treatment

Each experiment was performed in triplicate and replicated twice (n = 6). To represent the population of each pathogen throughout storage, results were expressed as colony forming units per millilitre (CFUs/mL) and consequently log transformed. Adhesion capability was expressed as means of adhesion index: (Nt/N0) × 100, where Nt represents the bacterial count (CFU) at specific time, and N0 represents the bacterial count (CFU) of the inoculum added to Caco-2 cells. Data were analysed using analysis of variance (ANOVA) in JMP Pro 17 software (SAS Institute Inc; Cary, NC, USA). Statistical significance was determined at *p* < 0.05.

A scatter matrix plot was generated using the same software to visualize the relationships between bacterial population reductions and the different antimicrobial compounds at various doses. Ellipses with a significance level of *p* = 0.05 were included to highlight potential groupings and correlations within the data.

GInaFIT 1.6 software was used for modelling the fit of the survival curves [57]. The Log-linear regression model is expressed by Equation (1):

(1) $$\log10\;\left(N\right)=\log10\;\left(N0\right)-\frac{t}{D}\;=\log10\;\left(N0\right)-\frac{\left(\mathrm{kmax}\times d\right)}{\mathrm{In}\;\left(10\right)}$$

where N represents the bacterial population at a specific time point (CFU/mL), N0 the initial bacterial population (CFU/mL), kmax the first order inactivation constant (h), and D the decimal reduction time (h). Alternatively, the Weibull model is represented by Equation (2):

(2) $$\log10\;\left(N\right)=\log10\;\left(N0\right)-{\frac{\left(t\right)}{\delta }}^{p}$$

where δ (h) is a scale parameter indicating the time required for the first decimal reduction, and p is a shape parameter that describes the curve’s concavity or convexity. The performance of these models was assessed using the root mean square error (RMSE) and the adjusted correlation coefficient (R^2^-adj), with the model having the lowest RMSE considered the best fit for the inactivation curve. To obtain the adhesion capability, results were expressed as means of Nt/N0, where Nt represents the bacterial count (CFU) at a specific time, and N0 represents the bacterial count (CFU) of the inoculum added to Caco-2 cells. All results were expressed as the mean ± standard deviation (SD).

## 4. Conclusions

This study investigated the effect of natural compounds on the survival and pathogenic potential of acid-adapted and non-acid-adapted *S.* Enteritidis and *E. coli* O157:H7 in orange juice. Based on our results, nisin (2 mL/L) and coumaric acid (0.5 g/L) were the most effective antimicrobials in reducing *S.* Enteritidis and *E. coli* O157:H7 populations in orange juice, respectively, even when the pathogens had been previously adapted to acid. Acid-adapted and non-acid-adapted *S.* Enteritidis showed a rapid reduction with nisin, reaching nearly a 5 and 1.5 log decrease after 30 h, respectively. Meanwhile *E. coli* O157:H7 experienced a similar reduction with coumaric acid after 48 h. These findings suggest that both compounds could be promising alternatives for controlling these pathogens in unpasteurized orange juice. Nevertheless, the acid-adapted *E. coli* O157:H7 showed great survival and pathogenic potential, which could represent a risk for public health and food safety. This study highlights the importance of considering the acid adaptation of pathogens when designing food preservation strategies and underscores the potential of synergistic combinations of antimicrobials to overcome cross-resistance. Future research should explore these synergistic mechanisms, optimize the doses of the compounds, and evaluate their efficacy in other matrices, such as other fruit juices.

## Figures and Tables

**Table 1:** Bacterial population reductions of non-acid-adapted *S.* Enteritidis and *E. coli* O157:H7 after 48 h at 4 °C in orange juice with different antimicrobial compounds at different doses.

	Bacterial Population Reduction (Log_10_ CFU/mL)	
	*S.* Enteritidis	*E. coli* O157:H7
OJ	0.5 ± 0.1	0.1 ± 0.1
OJ-0.25 mL/L citral	3.4 ± 0.2 b	0.3 ± 0.1 b
OJ-0.5 mL/L citral	4.8 ± 0.1 a	3.9 ± 0.6 a
OJ-0.05 g/L coumaric acid	1.0 ± 0.0 d	0.2 ± 0.1 c
OJ-0.1 g/L coumaric acid	1.3 ± 0.2 c	0.3 ± 0.1 b
OJ-0.25 g/L coumaric acid	2.1 ± 0.1 b	0.5 ± 0.1 b
OJ-0.5 g/L coumaric acid	4.2 ± 0.2 a	1.6 ± 0.0 a
OJ-0.5 mL nisin	1.3 ± 0.3 c	0.1 ± 0.1 b
OJ-1 mL/L nisin	2.2 ± 0.4 b	0.3 ± 0.2 ab
OJ-2 mL/L nisin	3.9 ± 0.5 a	0.4 ± 0.1 a
OJ-4 mL/L nisin	4.3 ± 0.1 a	0.4 ± 0.2 a

OJ: orange juice. Different letters represent significant differences between doses of the same antimicrobial compound according to analysis of variance (ANOVA) and Tukey’s Test (*p* < 0.05).

**Table 2:** Physicochemical characteristics of orange juice with and without antimicrobial compounds.

	pH	TSS (°Brix)	TTA (g Citric Acid/L)	a_w_	TPC (mg Gallic Acid/mL)	Antioxidant Capacity (mg Ascorbic Acid/L)	
						DPPH	FRAP
OJ	3.51 ± 0.02 a	11.6 ± 0.1 a	7.04 ± 0.26 a	1.000 ± 0.002 a	528.39 ± 25.22 a	1200.17 ± 3.85 a	977.98 ± 27.55 a
OJ-1 mL/L nisin	3.44 ± 0.01 a	11.7 ± 0.1 a	8.18 ± 0.12 a	0.993 ± 0.001 a	478.83 ± 10.13 a	1180.77 ± 5.52 a	986.88 ± 22.42 a
OJ-2 mL/L nisin	3.37 ± 0.02 a	11.7 ± 0.0 a	8.44 ± 0.09 a	0.994 ± 0.001 a	555.26 ± 22.73 a	1223.65 ± 42.11 a	981.54 ± 9.33 a
OJ-0.25 mL/L citral	3.66 ± 0.10 a	11.8 ± 0.2 a	7.02 ± 0.28 a	0.994 ± 0.001 a	655.56 ± 37.88 a	1304.31 ± 31.47 a	942.76 ± 16.34 a
OJ-0.50 mL/L citral	3.76 ± 0.03 a	11.8 ± 0.1 a	6.98 ± 0.22 a	0.988 ± 0.006 a	582.13 ± 27.57 a	1365.06 ± 4.92 a	950.94 ± 38.84 a
OJ-0.25 mL/L coumaric acid	3.70 ± 0.14 a	11.8 ± 0.0 a	7.32 ± 0.13 a	0.996 ± 0.001 a	782.03 ± 98.76 a	1300.74 ± 19.39 a	995.77 ± 15.13 a
OJ-0.50 mL/L coumaric acid	3.66 ± 0.02 a	11.6 ± 0.1 a	7.28 ± 0.47 a	0.997 ± 0.003 a	997.28 ± 65.74 a	1377.31 ± 1.77 a	985.1 ± 20.36 a

TSS = Total Soluble Solids, TTA = Titratable Acidity. TPC = Total Phenolic Content. DPPH = 2,2-diphenyl-1-picrylhydrazyl. FRAP = Ferric Reducing Antioxidant Power. Values are means of triplicates (n = 3) ± standard deviations. Means with different lowercase letters in the same columns indicate significant differences (*p* < 0.05) according to the LSD test.

**Table 3:** Statistical indices of predictive models estimating acid-adapted and non-acid-adapted *S.* Enteritidis and *E. coli* O157:H7 in orange juice with different antimicrobial compounds stored at 4 °C.

		Acid-Adapted *S.* Enteritidis	Non-Acid-Adapted *S.* Enteritidis	Acid-Adapted *E. coli* O157:H7	Non-Acid-Adapted *E. coli* O157:H7
Linear model					
OJ-1 mL/L nisin	RMSE	0.464	0.190	*	*
	R^2^-adj	0.760	0.988	*	*
OJ-2 mL/L nisin	RMSE	0.277	0.807	*	*
	R^2^-adj	0.963	0.859	*	*
OJ-0.25 mL/L citral	RMSE	0.357	0.593	0.072	0.318
	R^2^-adj	0.955	0.916	0.672	0.891
OJ-0.5 mL/L citral	RMSE	0.318	0.824	0.309	0.337
	R^2^-adj	0.970	0.836	0.957	0.965
OJ-0.25 g/L coumaric acid	RMSE	0.319	0.592	*	0.181
	R^2^-adj	0.947	0.883	*	0.958
OJ-0.5 g/L coumaric acid	RMSE	0.169	0.411	0.137	0.202
	R^2^-adj	0.992	0.954	0.929	0.987
Weibull model					
OJ-1 mL/L nisin	RMSE	0.470	0.511	*	*
	R^2^-adj	0.753	0.912	*	*
OJ-2 mL/L nisin	RMSE	*	0.931	*	0.094
	R^2^-adj	*	0.812	*	0.624
OJ-0.25 mL/L citral	RMSE	0.195	*	0.069	0.206
	R^2^-adj	0.986	*	0.693	0.954
OJ-0.5 mL/L citral	RMSE	0.345	*	0.280	0.306
	R^2^-adj	0.965	*	0.965	0.971
OJ-0.25 g/L coumaric acid	RMSE	0.300	0.516	*	0.172
	R^2^-adj	0.953	0.911	*	0.962
OJ-0.5 g/L coumaric acid	RMSE	0.137	*	0.119	0.204
	R^2^-adj	0.995	*	0.946	0.987

OJ: orange juice. RMSE: root mean squared error. R^2^-adj: coefficient of determination adjusted. *: data did not fit the predictive model.

**Table 4:** Kmax values obtained from the fitting of the linear model to survival curves of acid-adapted and non-acid-adapted *S*. Enteritidis and *E. coli* O157:H7 in orange juice with different antimicrobial compounds stored at 4 °C.

	Acid-Adapted *S.* Enteritidis	Non-Acid-Adapted *S.* Enteritidis	Acid-Adapted *E. coli* O157:H7	Non-Acid-Adapted *E. coli* O157:H7
OJ-1 mL/L nisin	0.11 ± 0.01	0.22 ± 0.00	*	*
OJ-2 mL/L nisin	0.18 ± 0.01	0.26 ± 0.02	*	*
OJ-0.25 mL/L citral	0.21 ± 0.01	0.25 ± 0.01	0.01 ± 0.00	0.12 ± 0.01
OJ-0.5 mL/L citral	0.33 ± 0.01	0.43 ± 0.04	0.21 ± 0.01	0.22 ± 0.01
OJ-0.25 g/L coumaric acid	0.17 ± 0.01	0.29 ± 0.02	0.02 ± 0.00	0.11 ± 0.00
OJ-0.5 g/L coumaric acid	0.34 ± 0.01	0.43 ± 0.02	0.06 ± 0.00	0.22 ± 0.00

OJ: orange juice. Kmax: first order inactivation rate constant. ±: represent the standard error. *: data did not fit the predictive model.

**Table 5:** Kinetic parameters (δ and p) obtained from the fitting of the Weibull model to survival curves of acid-adapted and non-acid-adapted *S*. Enteritidis and *E. coli* O157:H7 in orange juice with different antimicrobial compounds stored at 4 °C.

		Acid-Adapted *S.* Enteritidis	Non-Acid-Adapted *S.* Enteritidis	Acid-Adapted *E. coli* O157:H7	Non-Acid-Adapted *E. coli* O157:H7
OJ-1 mL/L nisin	δ	22.69 ± 4.16	3.48 ± 1.11	*	*
	p	1.07 ± 0.25	0.60 ± 0.07	*	*
OJ-2 mL/L nisin	δ	-	7.80 ± 3.50	*	78.83 ± 12.18
	p	-	0.79 ± 0.20	*	2.16 ± 0.61
OJ-0.25 mL/L citral	δ	17.85 ± 0.80	*	119.67 ± 29.29	26.07 ± 1.33
	p	1.53 ± 0.07	*	1.29 ± 0.31	1.63 ± 0.13
OJ-0.5 mL/L citral	δ	7.87 ± 1.29	*	14.14 ± 1.28	8.25 ± 1.22
	p	1.05 ± 0.13	*	1.23 ± 0.10	0.84 ± 0.07
OJ-0.25 g/L coumaric acid	δ	16.69 ± 1.57	2.33 ± 1.07	*	23.46 ± 1.41
	p	1.23 ± 0.11	0.53 ± 0.09	*	1.19 ± 0.09
OJ-0.5 g/L coumaric acid	δ	5.18 ± 0.38	*	38.37 ± 1.28	10.85 ± 0.82
	p	0.86 ± 0.04	*	1.41 ± 0.13	1.03 ± 0.05

OJ: orange juice. ±: represents the standard error of each parameter. δ: time for the first decimal reduction (h). p: shape parameter (dimensionless). *: data did not fit the predictive model.

**Table 6:** Adhesion capability of acid-adapted and non-acid-adapted *S*. Enteritidis and *E. coli* O157:H7 in orange juice with different antimicrobial compounds stored at 4 °C.

			Adhesion Index
*S.* Enteritidis	Acid-adapted	Orange juice	10.6 ± 13.5 a
	Non-acid-adapted	Orange juice	6.5 ± 1.3 a
	Acid-adapted	Orange juice with 1 mL/L nisin	2.4 ± 1.8 b
	Non-acid-adapted	Orange juice with 1 mL/L nisin	0.0 ± 0.0 b
*E. coli* O157:H7	Acid-adapted	Orange juice	40 ± 50.6 a
	Non-acid-adapted	Orange juice	0.0 ± 0.0 a
	Acid-adapted	Orange juice with 0.5 g/L cumaric acid	14.1 ± 2.5 a
	Non-acid-adapted	Orange juice with 0.5 g/L cumaric acid	12.1 ± 1.3 a

Values are means of six repetitions ± standard deviations. Means with different lowercase letters indicate significant differences (*p* < 0.05) between acid-adapted and non-acid-adapted bacteria according to the LSD test.

## Data Availability

The original contributions presented in this study are included in the article. Further inquiries can be directed to the corresponding author.
